# BayesTICS: Local temporal image correlation spectroscopy and Bayesian simulation technique for sparse estimation of diffusion in fluorescence imaging

**DOI:** 10.1017/S2633903X23000041

**Published:** 2023-02-27

**Authors:** Anca Caranfil, Yann Le Cunff, Charles Kervrann

**Affiliations:** 1 SERPICO Project-Team, INRIA Rennes, UMR144 CNRS Institut Curie, PSL Research, Sorbonne Université, Campus universitaire de Beaulieu, Rennes, France; 2 CeDRE Team, GDR UMR6290-CNRS, Faculty of Medicine, University of Rennes 1, Rennes, France; 3 Dyliss Team, Univ Rennes, CNRS, Inria, IRISA, UMR 6074, Campus de Beaulieu, Rennes, France

**Keywords:** Bayesian methods, diffusion, estimation, fluorescence microscopy, temporal correlation spectroscopy

## Abstract

The dynamics and fusion of vesicles during the last steps of exocytosis are not well established yet in cell biology. An open issue is the characterization of the diffusion process at the plasma membrane. Total internal reflection fluorescence microscopy (TIRFM) has been successfully used to analyze the coordination of proteins involved in this mechanism. It enables to capture dynamics of proteins with high frame rate and reasonable signal-to-noise values. Nevertheless, methodological approaches that can analyze and estimate diffusion in local small areas at the scale of a single diffusing spot within cells, are still lacking. To address this issue, we propose a novel correlation-based method for local diffusion estimation. As a starting point, we consider Fick’s second law of diffusion that relates the diffusive flux to the gradient of the concentration. Then, we derive an explicit parametric model which is further fitted to time-correlation signals computed from regions of interest (ROI) containing individual spots. Our modeling and Bayesian estimation framework are well appropriate to represent isolated diffusion events and are robust to noise, ROI sizes, and localization of spots in ROIs. The performance of BayesTICS is shown on both synthetic and real TIRFM images depicting Transferrin Receptor proteins.

## Impact Statement

This paper presents an original Bayesian method to analyze fluorescent spots diffusing at the cell membrane and observed in time-lapse fluorescence microscopy. Unlike related temporal correlation spectroscopy methods, BayesTICS adapts locally and is robust to low signal-to-noise ratios and sizes of regions of interest.

## 1. Introduction

In cell biology, diffusion measurements are commonly used to compare several compartments within a cell or in different cells (e.g., neuronal synapses or HeLa cells). At the scale of a single cell, free diffusion of proteins (i.e., molecules undergoing Brownian motion) often corresponds to the main mode of molecular transport. The analysis of intracellular diffusion provides information about both the dynamics of the proteins and the cellular medium in which they evolve. Several techniques have been developed to quantify the dynamics of fluorescently-tagged proteins (e.g., Green Fluorescent Proteins [GFP]) in live cell imaging and to estimate diffusion coefficients from fluorescence microscopy data, that were adapted to different purposes. The two main types of techniques are single-particle techniques and ensemble measurement techniques, both allowing for the analysis of molecular dynamics, each having their advantages and limitations as described below.

Single-particle tracking (SPT) approaches are particularly recommended when the experimental setup is available, as the trajectory of a single molecule provides a quantitative description of motion in space and time. Given tracks obtained by nearest neighborhood algorithms or more sophisticated tracking methods^(^^1^ ^)^, the mean-square displacement (MSD) of tracks is generally used to interpret and detect free diffusion, confined diffusion, and directed flow^(^^2^ ^)^. The theoretical limits of SPT, in particular for confined diffusion, have been established in Ref. (3). SPT-based approaches require labeling single molecules with suitably high signal/noise marker particles, as well as high-speed and high-sensitivity microscopes. Tracking errors due to imperfect algorithms can also limit the applicability of this technique.

Fluorescence correlation approaches are valuable techniques that provide quantitative information on both large and small-scale intracellular dynamics without individual object tracking. In the original fluorescence correlation spectroscopy (FCS) method^(^^4^ ^–^^6^ ^)^, temporal fluctuations of fluorescent molecules in a region of interest (ROI) are used to measure the local concentration of the observed population. A correlation method is used to analyze 1D signals of temporal fluctuations. The main drawback of FCS is related to the photobleaching of the fluorescently-tagged molecules that limits the acquisition time and, thus, the applicability to fast dynamics. A related approach consists in analyzing the fluorescence recovery after photobleaching (FRAP)^(^^7^ ^)^ in a specified area by a high-intensity laser pulse and is used to quantify two-dimensional lateral diffusion and protein binding. The fluorescence recovery curves are fitted to a theoretical model by using a nonlinear least squares algorithm^(^^8^ ^)^. However, reliable estimation of the parameters requires several experimental curves that might introduce numerical and experimental repetition errors. The major limitations of both FCS and FRAP are related to strong sensitivity to the signal-to-noise ratio (SNR) and the need for spatial and temporal stationarity of dynamics in the ROI.

Another approach that is derived from fluorescence correlation techniques and overcomes some of FCS limitations is temporal image correlation spectroscopy (TICS)^(^^9^ ^)^. With TICS, one can measure the concentration of molecules in the image as well as dynamical properties such as the characteristic time of diffusion. TICS is based on the same principles as FCS but exploits 2D temporal signals instead of 1D temporal signals, and can detect diffusion and flow at the same time. Spatio-temporal image correlation spectroscopy (STICS)^(^^10^ ^)^ extended the applicability of TICS to recover direction flow of moving molecules. Other derived methods were developed for specific purposes, such as spatio-temporal image cross-correlation spectroscopy (STICCS)^(^^11^ ^)^ that can detect and characterize the dynamics of two species in the ROI, reciprocal space image correlation spectroscopy (kICS)^(^^12^ ^)^ that solves the issue of sensitivity to photobleaching and blinking of the fluorescent molecules of STICS or, more recently, pair correlation function FCS (pCF)^(^^13^ ^)^ that is able to detect barriers of diffusion. While a wide range of applications is covered by these correlation-based methods, they still require a low concentration of molecules in the region of focus and a temporal stationarity of the fluorescence signals^(^^10^ ^,^^14^ ^)^, which is not always satisfied in experimental data.

All the aforementioned methods are either computationally demanding or come with a high sensitivity to parameters such as SNR or ROI size. Moreover, standard correlation-based methods require a previous knowledge of the point spread function (PSF) that is not always available, and need a minimum window size, which makes it impossible to estimate the diffusion coefficient on very small ROIs. Thus, analyzing diffusion in small inhomogeneous regions of the cell from regular 2D fluorescent microscopy data is still a real challenge.

In this paper, we address the issue of estimating locally constant but spatially varying diffusion coefficients of fluorescently-tagged membrane proteins close to the plasma membrane (PM). Experimental data is acquired by total internal reflection fluorescence (TIRF) microscopy, a technique vastly used for imaging protein dynamics within thin layers^(^^15^ ^,^^16^ ^)^. We propose two models of the autocorrelation function of the 2D temporal fluorescent signal and a Bayesian framework (approximate Bayesian computation (ABC)^(^^17^ ^)^) for robustly estimating the local diffusion coefficient. We show that the proposed method complements previous techniques by providing distinct advantages of local analysis over TICS counterparts on ROIs. Moreover, the method does not rely on a temporal stationarity hypothesis of the fluorescent signal or an accurate measurement of PSF of the imaging system. Unlike previous methods based on nonlinear model fitting^(^^18^ ^)^, BayesTICS is based on the simulation of time-correlation signals and provides the posterior distribution of diffusion coefficient that can be exploited to analyze changes in dynamics, or in the local environment of the membrane.

This paper is organized as follows. In the next section, we describe the diffusion and image models with initial conditions, the closed-form solution and approximations, including the ABC-based algorithm to estimate local diffusion coefficients from time-correlation signals computed over ROIs. In the experimental section, we demonstrate the robustness of BayesTICS to SNRs on simulated and real images depicting Transferrin Receptor (TfR) dynamics (labeled with pHluorin, a pH-sensitive derivative of GFP) at the PM during the late steps of exocytosis, that is, diffusion in the PM after vesicle fusion. We evaluate the sensitivity to both nuisance parameters (e.g., size of ROIs) and the algorithm parameters, and we assess the accuracy of BayesTICS on simulated data. Our approach further extends the capability of correlation spectroscopy methods and provides, to our knowledge, a new tool for the quantitative characterization of molecular dynamics in living cells.

## 2. Method

### 2.1. Diffusion model and image generation

Diffusion of transmembrane proteins is mainly modeled by lateral diffusion in the membrane^(^^19^ ^)^. This phenomenon may be described by Fick’s second law^(^^20^ ^)^:
(1)

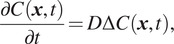

where 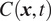 denotes the concentration of molecules at time 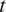 and at location 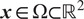 , 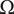 being the ROI in the image domain, and 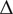 is the Laplacian operator. In our modeling framework, the diffusion coefficient 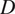 in (1) is assumed to be constant over 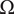 . In addition, we assume that all the proteins are concentrated at the center 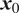 of 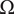 , at initial time 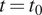 , that is 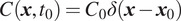 where 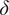 is the Kronecker symbol and 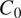 is the initial concentration of molecules. With the aforementioned initial conditions, the closed-form solution is well established and given by^(^^21^ ^,^^22^ ^)^:
(2)

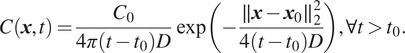



Define 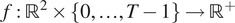 , and denote 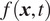 the fluorescence intensity at time 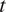 and spatial position 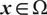 . The fluorescence intensity is assumed to be proportional (with factor 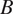 ) to the convolution (denoted 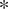 ) of the microscopic number density (or concentration) 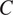 and the instrumental “PSF” 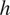 :
(3)

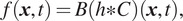

where 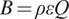 , 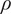 is the efficiency of the instrument to collect photons, 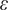 is the molecular absorption coefficient, and 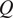 is the quantum yield of the fluorophore. If we assume that the PSF is approximated with a 2D Gaussian function with an isotropic bandwidth 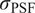 in the lateral direction, we get (see Refs. 18,23) and Supplementary Appendix A):
(4)

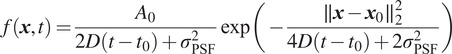

with 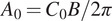 .

### 2.2. Local temporal image correlation spectroscopy

In this section, we describe our correlation-based method, inspired from FCS, to compute local diffusion in fluorescence images. For this purpose, we need to compute the temporal autocorrelation of images to capture temporal fluctuations of intensities.

#### 2.2.1. Uniform background

First, we are interested in locally estimating the diffusion of an isolated spot over a uniform background (i.e., for a long enough sequence, the spatiotemporal average of 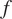 is zero). In that case, the process associated with the image sequence is not stationary. Accordingly, the autocorrelation function is real-time dependent and not only delay-dependent. Hence, we use the following expectation formula for the autocorrelation function:
(5)

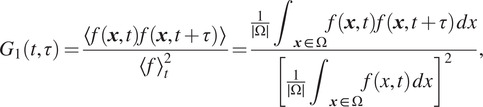

where 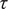 is the temporal lag and denote the spatial average. By substituting 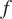 with (4) in (5), we get (see details in Supplementary Appendix B):
(6)

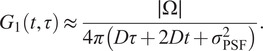

Note that if we consider the inverse of the autocorrelation function, 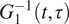 can be approximated as a linear function of unknown parameters 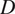 and 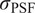 (see details in Supplementary Appendix B):
(7)

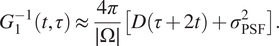



#### 2.2.2. Nonuniform and cluttered background

In order to account for nonuniform background and the potential presence of neighboring spots diffusing in the ROI 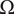 , let us assume that the spatiotemporal average of 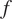 is nonzero. Hence, we consider the following expectation formula for the intensity fluctuation autocorrelation function:
(8)

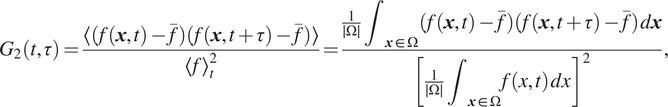

where 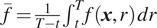 . By replacing 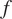 in (8) with the expression given in (4), we obtain (see details in Supplementary Appendix C):
(9)

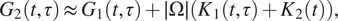

where

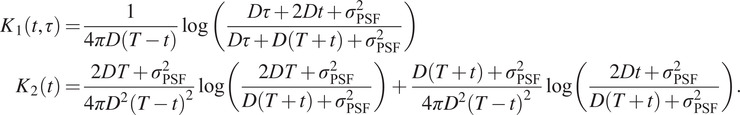

Model 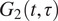 can then be considered as an extension of the model 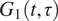 . In (6) and (9), the ROI size 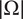 is a multiplicative factor and can be interpreted as a scaling parameter. We shall see that the ROI size does not influence the estimation of 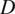 in our experiments provided there is a single spot in the ROI. Unlike (6), the total time of observation 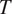 appears in 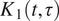 and 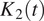 and may thus influence the estimation of 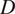 . Nevertheless, this impact is minimal for 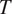 large enough, as one has

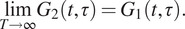

Finally, the extended model 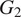 can be interpreted as an “out of equilibrium” state of the observed system, that allows us to take into account perturbations such as noisy data, nonuniform background, or other spots diffusing in the image.

### 2.3. Bayesian estimation of model parameters

Model 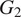 , as given by (9), depends on the values of 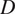 and 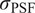 in a highly nonlinear fashion. As a result, the estimation of the involved parameters performed by maximizing the underlying likelihood (or minimizing the corresponding data fidelity term) may be very complex or even not tractable. Nevertheless, we can resort by applying a computational Bayesian approach described in what follows.

#### 2.3.1. Principles of ABC

The ABC method^(^^17^ ^)^ relies on stochastic simulation that generates samples and selects those that follow the posterior distribution. This approach allows us to compute both the maximum a posteriori estimator and the a posteriori expectation from the selected samples.

Formally, let us assume that a given discrete observation 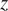 is generated from a model with parameters 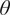 whose prior is denoted by 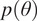 . The posterior distribution of interest is defined by 
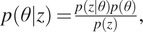
 where 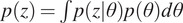 . The success of the rejection ABC method^(^^17^ ^)^ depends on the fact that the underlying stochastic process is easy to simulate for a given set of parameters. The ABC procedure can be summarized as below:

1. Generate 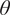 from the prior distribution 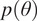 ;
2. Simulate 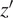 from the model with parameter 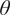 ;
3. Compute the distance 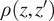 between 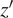 and 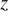 ;
4. Accept 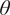 with probability 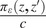 and return to 1.

Here 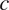 is a constant chosen to guarantee that 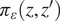 defines a probability:

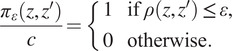

The observation 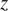 and the simulation 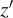 are real-valued arrays of matching dimension and 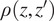 evaluates the distance between them.

As mentioned above, this approach requires the design of a suitable metric 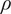 , as well as the choice of an adapted value for 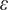 . The choice of 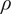 depends on the given problem. However, standard metrics, such as the 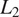 norm, are used in a significant number of problems. As for the choice of 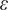 , one may note that, when 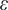 tends to 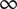 , the accepted samples come from the prior, while when 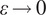 , the accepted samples follow the target posterior distribution 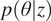 . Therefore, the choice of 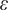 reflects the balance between computability and accuracy. For a given 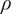 and 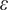 , accepted samples are independent and identically distributed from 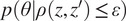 .

The next step is to compute posterior expectation defined as 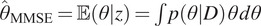 , which is known as the minimum mean square error (MMSE) estimator. The simplest way to compute the MMSE estimator is to draw 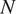 samples 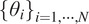 , from 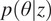 using the algorithm above and compute the empirical averages 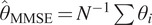 . The samples can also be exploited to compute the posterior distribution for which the maximum mode equals the maximum a posteriori (MAP) estimator 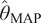 . There are several advantages to this rejection method, among them the fact that they are usually easy to code, and they generate independent samples.

#### 2.3.2. ABC algorithm and implementation details

In our context, the observation 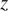 is the autocorrelation (5) or (8) approximated in the discrete setting from the fluorescent intensities 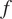 , in a given ROI 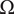 , as follows:
(10)

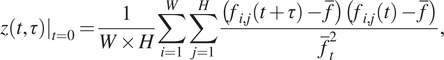

where 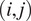 denotes a pixel location in 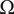 , 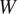 and 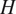 are the width and height of the ROI ( 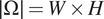 ), 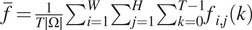 , and 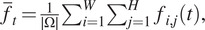 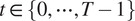 . The simulated sample 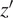 is the autocorrelation simulated from the model (6) and (9) with parameter 
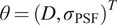
 .

We implemented the ABC rejection-based method with 0–1 cut-off and the distance 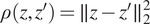 (see Algorithm 1). The algorithm takes as input an image sequence, also denoted 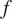 , and computes 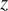 as explained above. Then, it uses the ABC procedure to compute the estimates 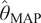 and 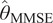 .

Algorithm 1: BayesTICS algorithm

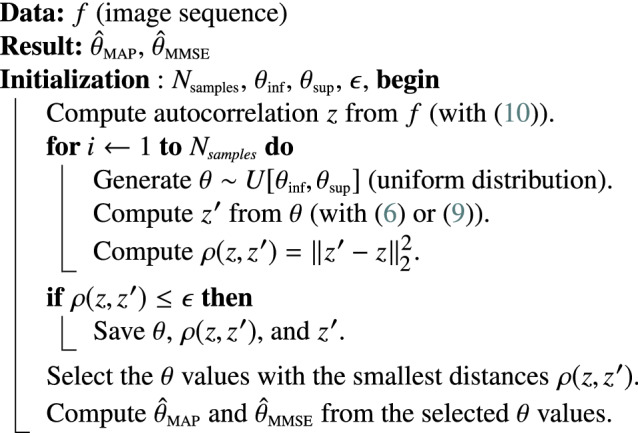

We now discuss how the parameters of BayesTICS can be selected. The prior distribution of both parameters 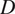 and 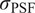 is assumed to be uniform on intervals depending on the underlying biological process. Typically, 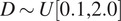 and 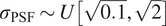 . The number 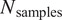 is set to 100, 000 samples. The cut-off value 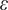 is set so as to accept 1 to 5% of the samples (about the best 1000 samples) which serve to compute the posterior distribution, 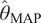 , and 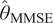 .

The algorithm is relatively fast as each sample 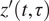 from (6) or (9) takes about 0.001 s to generate. Moreover, all the simulated samples in a ROI are independent and can be generated in parallel. This means that the processing of multiple ROIs in an image sequence can be performed very efficiently.

## 3. Experimental Results

**Figure 1.**
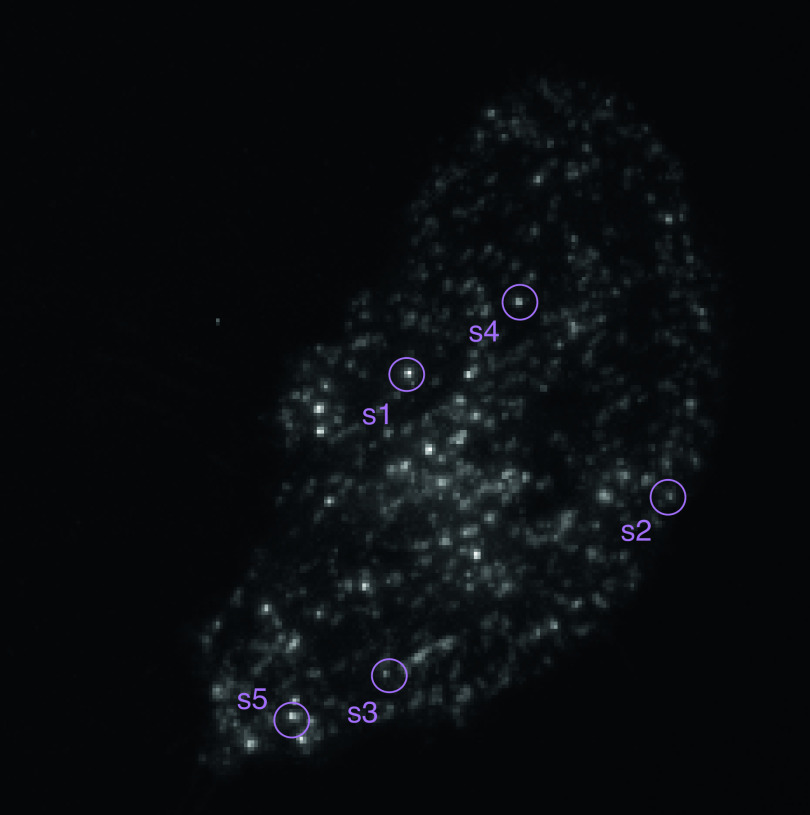
Microscopy image depicting diffusing pHluorin-tagged spots at the PM observed in TIRF microscopy (courtesy of PICT facility, UMR144-CNRS Institut Curie).

In this section, we evaluate the performance of BayesTICS on synthetic and real images depicting multiple diffusing spots at the PM observed in TIRF microscopy as illustrated in Figure 1. We applied the BayesTICS algorithm to compute the MAP and MMSE estimators of the diffusion coefficient by considering the two models (6) and (9), respectively. The ROI size depends on the spot size and here is set to 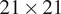 pixels windows from the analysis of real images. Meanwhile, the temporal series should be long enough to observe stationary fluctuations. In our experiments, 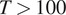 time points. As these choices may involve some arbitrariness, we assessed the sensitivity to 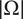 and 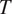 , as well as the position of the spot of interest in the ROI. In what follows, we show that changes in these parameters lead to essentially similar results and demonstrate the robustness of BayesTICS with respect to noise levels and cluttered background (i.e., presence of multiple spots in a ROI). We focus on the estimation of 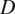 which is the parameter of interest.

### 3.1. Evaluation on simulated data

**Figure 2.**
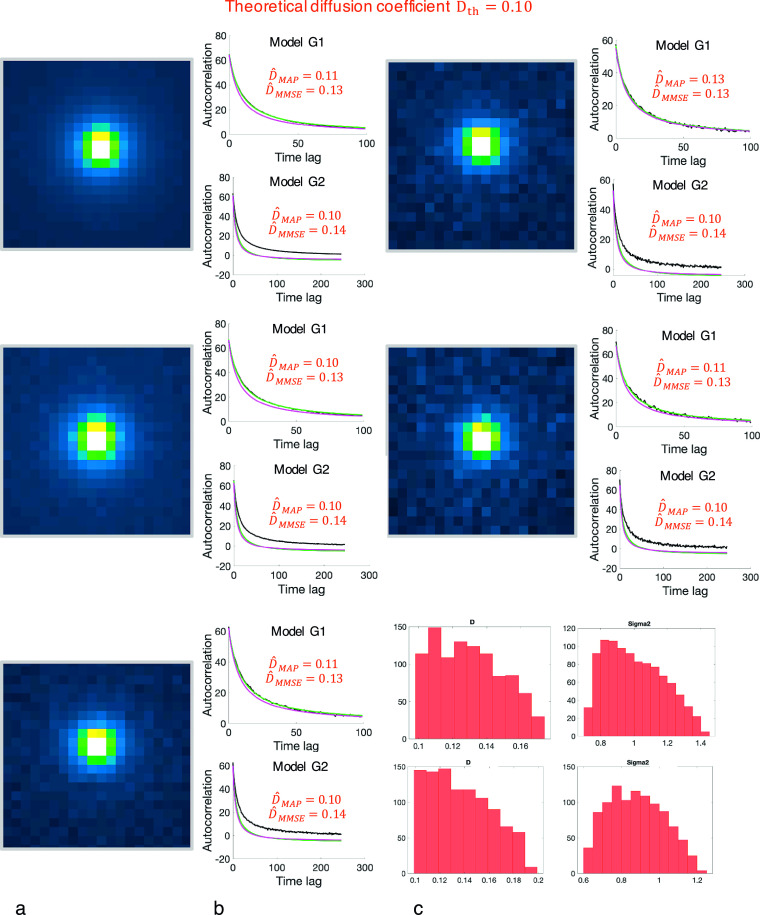
Robustness of the BayesTICS method to noise level with a fixed window size. Five different ROIs with increasing amount of noise (from top to bottom, and from left to right) were simulated. (a) The ROIs at time 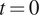 are extracted from five simulated sequences composed of 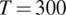 frames ( 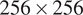 pixels images) and depicting 2D diffusing spots with a theoretical diffusion coefficient 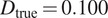 pixel/frame. The size of ROIs is set to 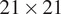 pixels and the spots are located at the center of each ROI. (b) The autocorrelation versus time lag plot is shown for 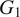 and 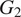 models. Each plot shows the observed autocorrelation (black curve), and two autocorrelation samples generated from the 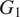 and 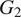 models with the 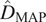 and 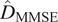 parameters (green and magenta curves). (c) The estimated posterior distributions are displayed for 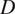 and 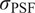 for the ROI with intermediate noise level.

First, in order to test the robustness of BayesTICS to noise, we simulated six image sequences depicting an isolated diffusion spot in a ROI 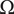 of 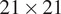 pixels, with a diffusion coefficient equal to 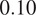 pixel/frame. The image intensities were then corrupted with Gaussian white noise with different standard deviations yielding image sequences with different SNRs. Figure 2 shows the 2D images at time 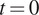 (left) extracted from the six image sequences and the autocorrelation plots (middle and right). We reported the results of BayesTICS, that is the posterior distributions, 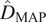 , and 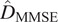 for the two autocorrelation models 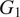 and 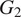 , respectively. The results suggest that the performance of estimators 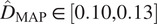 pixel/frame and 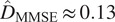 pixel/frame for 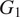 , and 
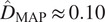
 pixel/frame and 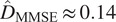 pixel/frame for 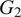 , are hardly influenced by the amount of noise in the images. We observe that the MMSE values are slightly higher than the true diffusion coefficient ( 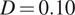 pixel/frame) for both models.

**Figure 3.**
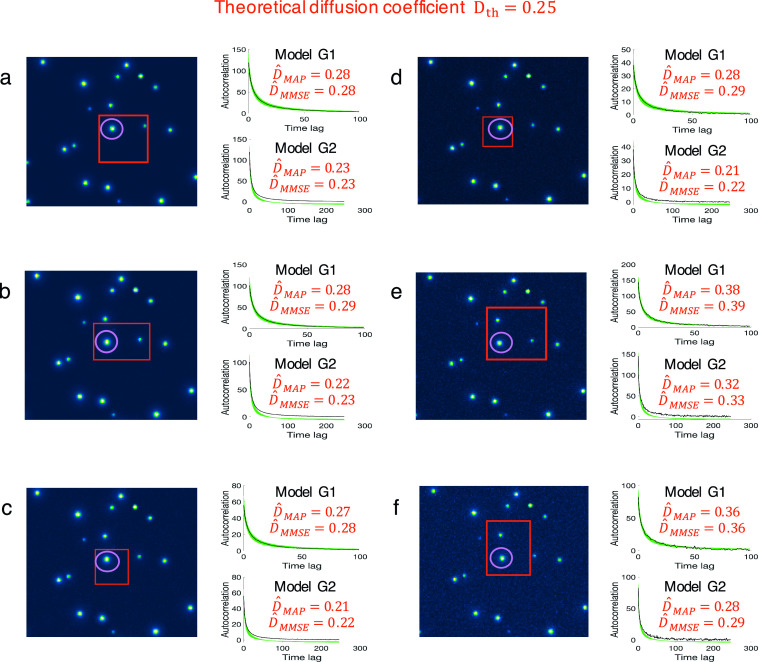
Robustness of the BayesTICS method to noise level with variable spot position, and window size. (a-f) Six different cases with varying noise level, spot position, and window size were tested. They were extracted from six simulated sequences of 2D diffusing spots, with 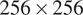 pixels window size, total length of 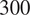 frames, a theoretical diffusion coefficient of 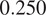 pixels/frame. For each case, the *z*-projection of the maximum intensity of the simulated stack and the autocorrelation versus time lag plot is shown. The spot of interest is market in purple, and can be anywhere in the ROI. Other spots can be in the ROI, diffusing or not. The size of the ROI has the following values (from a to f): 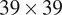 , 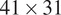 , 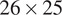 , 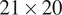 , 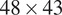 , 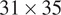 pixels. Each plot shows the computed autocorrelation from the data (dark gray), generated autocorrelations for the BayesTICS method (green). The two estimates 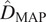 and 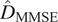 for the diffusion coefficient are given on the corresponding plots.

Furthermore, we evaluated the influence of the ROI size, the spot position in ROIs, and the amount of noise in ROIs. We analyzed six different ROIs shown in Figure 3. The spot of interest (red circle) is not located at the center of the ROI. The size of the ROI varies from small regions to large square/rectangle windows which may contain background and additional spots. The SNR changes from low values (upper-left image in Figure 3) to high values (lower-right image in Figure 3). In this experiment, we obtained 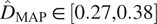 pixel/frame and 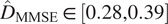 pixel/frame for 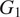 , and 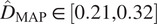 pixel/frame and 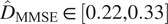 pixel/frame for 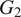 , while 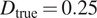 pixel/frame. Both MAP and MMSE estimators display a certain variance and inaccuracy that is due to the background environment in the ROI. Supplementary Figures 1–3 show that the change in ROI size or in spot position do not influence significantly the estimation, but a combined effect with the background clutter in the ROI can potentially diminish the accuracy of the estimation. Interestingly, both MAP and MMSE estimators from model 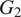 are more accurate than the estimation from 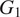 , suggesting that model (9) is better suited for spots in complex environments than model (6).

### 3.2. Experiments on real data: TfR molecules diffusion at different locations at the plasma membrane

We assessed the BayesTICS algorithm on real data total internal reflection fluorescence microscopy (TIRFM) 2D image sequences depicting TfR proteins tagged with pHluorin in M10 cells, at the late steps of exocytosis, that is when the vesicles fuse with the PM. TfR is a transmembrane protein known to diffuse at the PM, and serves here as a reference biological model. PHluorin is pH-sensitive and is helpful here to determine the accurate time-point when the fusion starts. This probe was already used in previous studies^(^^23^ ^,^^24^ ^)^. We considered 2D TIRFM sequences composed of 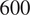 images of size 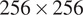 pixels (see Figure 1), acquired with a frame rate of 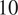 frames/s.

**Figure 4.**
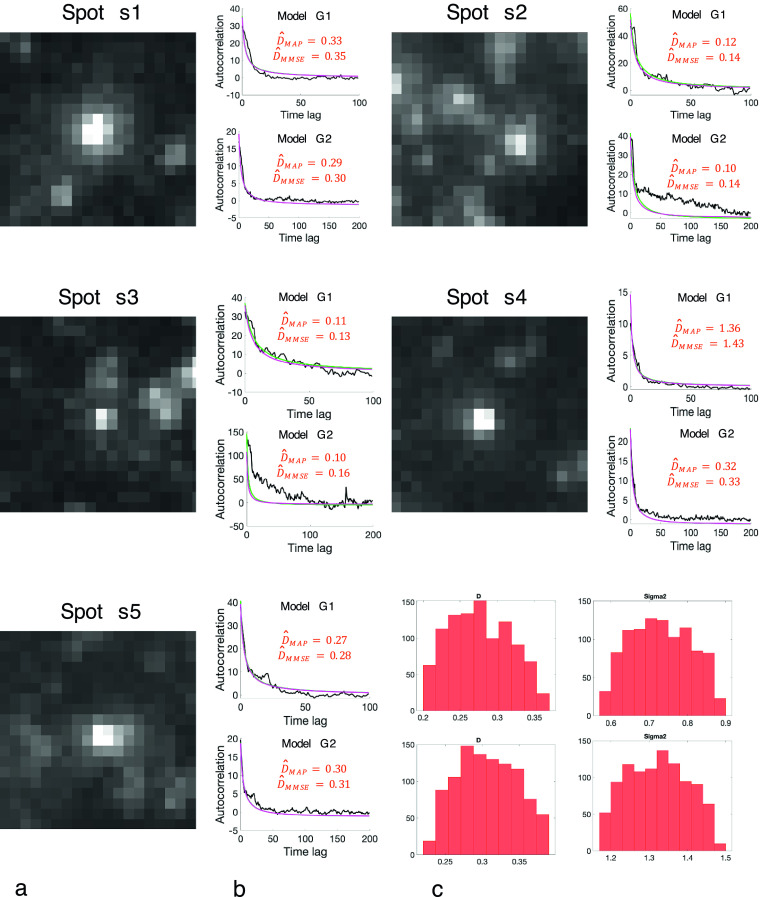
Evaluation of BayesTICS on real TIRF image sequences depicting TfR proteins tagged with pHluorin (pH-sensitive probe) in M10 cells. (a) Five 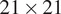 pixels ROIs were selected (left). (b) The autocorrelation versus time lag plot are shown for 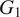 and 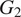 models. Each plot shows the observed autocorrelation (black curve), and two autocorrelation samples generated from the 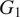 and 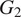 models with the 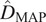 and 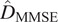 parameters (green and magenta curves). (c) The estimated posterior distributions are displayed for 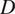 (left) and 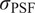 (right) for Spot s5.

Local diffusion events are first detected at the PM with an appropriate algorithm^(^^18^ ^,^^23^ ^)^ (or manually selected by an user) and several temporal series of ROIs with the same size 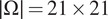 are automatically extracted as illustrated in Figure 4. The nonuniform fluorescent background in 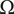 is estimated as the median of the intensity values, over 20 frames, and further subtracted to reduce bias in the estimation of diffusion. The initial time point 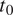 is automatically found in our experiments by detecting the frame in which the fluorescence intensity is maximal in the temporal sequence. For a given ROI, the frames with index 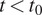 are discarded. The value of 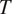 is set to 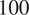 and 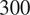 for computing 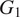 and 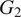 , respectively. As the background is subtracted from the last few dozen frames, 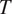 must be chosen as to keep the end of the sequence free of other diffusing spots. As long as this condition is satisfied, 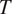 can be tuned according to the underlying diffusion speed. Finally, we assume that the images are corrupted by white Gaussian noise. A variance stabilizing transform (Generalized Anscombe transform^(^^24^ ^)^) is potentially applied to produce a normally distributed noise^(^^25^ ^)^.

We selected five ROIs (s1,s2, s3, s4, s5) in the image shown in Figure 1 which contain a diffusing spot and variable background. We applied BayesTICS to compute the posterior distributions of 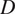 and 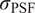 and the MAP and MMSE estimators by considering the two models 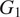 and 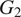 . In the present situation, the MAP and MMSE estimators ( 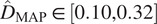 , 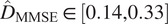 ) computed with the 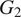 model are more consistent with those published in the literature^(^^18^ ^,^^23^ ^)^.

## 4. Conclusion

In this paper, we have proposed a novel method, named BayesTICS, to estimate local 2D diffusion observed in TIRF microscopy from temporal series of images acquired for typical temporal correlation measurements. BayesTICS was specifically designed for local estimation, as opposed to other methods such as FRAP or FCS that provide a global estimator for the entire image. We assume that the diffusion coefficient is constant in the region of interest, that is, for each diffusing spot, but not necessarily constant across the entire image. BayesTICS may be helpful to confirm if diffusion is homogeneous or heterogeneous across several spots. In our approach, the fluorescent intensity and a nonstationary initial diffusion model are used to compute the autocorrelation function of the sequence. This function is then used in a correlation-based Bayesian framework providing the posterior distribution of the diffusion coefficient. Two models are proposed: one for isolated diffusing spots without background ( 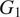 ), and one for nonisolated diffusing spots with crowded background ( 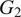 ). The efficiency and accuracy of the method are demonstrated on extensive simulated sequences, then applied to experimental data.

By applying the BayesTICS method to the diffusion of TfR molecules during the fusion of vesicles to the PM in exocytosis, we showed that this method is able to properly handle a wide variety of situations, yielding results that are consistent and in accordance with previously reported results. The results show that the apparent diffusion coefficient varies slightly between the different spots, which is helpful here to detect local variations in the environment of the diffusing spot. In Supplementary Figure 4, we illustrate BayesTICS in a complementary study focused on the interactions between TfR and Rab11 proteins that dissociate or diffuse close to the PM. Our method suggests that a small fraction of the detected Rab11 spots display apparent diffusion at the PM. These preliminary results need to be confirmed with alternative and complementary estimation techniques as currently investigated in an on-going project.

BayesTICS is a fast and simple-to-implement method for local diffusion quantification from TIRF microscopy data. Its main advantages are that it uses standard TIRF microscopy acquisition, and only needs a single sequence to estimate local diffusing with little preprocessing. This both reduces experimental setup costs and avoids extra numerical errors that come from repeating experiments and from complex pre or postprocessing. This method is also robust to variable SNRs, to the choice of the size of the analysis window, and to the location of the spot of interest in the ROI. Two limitations remain: the method requires long acquisition sequences (at least 100 frames for 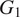 and 300 frames for 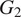 ), which is due to the correlation basis itself and, it is sensitive to dynamic interferences in the ROI, which could be improved by introducing the appropriate dynamic initial model to include other types of diffusion. While BayesTICS is currently designed to detect 2D diffusion only, its extension to 3D and, in particular, its application to 3D multi-angle TIRFM^(^^26^ ^)^ are envisioned, in order to better decipher the late steps of the exocytosis mechanisms.

## Supplementary material

For supplementary material accompanying this paper visit https://doi.org/10.1017/S2633903X23000041. click here to view supplementary material
