# Supplementary material for: BayesTICS: Local temporal image correlation spectroscopy and Bayesian simulation technique for sparse estimation of diffusion in fluorescence imaging
Source: Biol Imaging. 2023 Feb 27;3:e5. doi: 10.1017/S2633903X23000041 (PMC10936362; doi:10.1017/S2633903X23000041)
Supplement: Supplementary file 1 [file S2633903X23000041sup001.pdf]

Biological Imaging (2022), 1: 1–12
doi:10.1017/blg.2022.xx

CAMBRIDGE UNIVERSITY PRESS

RAPID COMMUNICATION

SUPPLEMENTARY INFORMATION

BayesTICS: Local temporal image correlation spectroscopy and Bayesian simulation technique for sparse estimation of diffusion in fluorescence imaging

Anca Caranfil[1,2], Yann Le Cunff[2,3] and Charles Kervrann[1]*

[1]Serpico Team-Project, Inria Rennes, UMR144-CNRS, Institut Curie, PSL Research, Sorbonne Universités, Campus universitaire de Beaulieu, 35042 Rennes Cedex, France
[2]CeDRE Team, GDR UMR6290-CNRS, Faculty of Medicine, University of Rennes 1, 2 avenue du Prof. Léon Bernard, CS 34317, 35043 Rennes cedex, France
[3]Dyliss Team, Univ Rennes, CNRS, Inria, IRISA, UMR 6074, Campus de Beaulieu, 35042 Rennes Cedex, France
*Corresponding author. Email: charles.kervrann@inria.fr

Received xx xxx xxxx

© The Authors(s) 2021. Published by Cambridge University Press. This is an Open Access article, distributed under the terms of the Creative Commons Attribution licence (http://creativecommons.org/licenses/by/4.0/), which permits unrestricted re-use, distribution, and reproduction in any medium, provided the original work is properly cited.

A. Spot diffusion model

The fluorescence intensity f is assumed is proportional (with factor B) to a convolution (denoted $*$) of the microscopic number density (or concentration) C and the instrumental point spread function h:

$$f(\boldsymbol{x}, t) = B\,(h * C)\,(\boldsymbol{x}, t), \tag{1}$$

where $B = \rho\varepsilon Q$, ρ is the efficiency of the instrument to collect photons, ε is the molecular absorption coefficient, and Q is the quantum yield of the fluorophore. We consider the PSF is approximated with a 2D Gaussian function with an isotropic bandwidth σ_{PSF} in the lateral direction. Without loss of generality, we assume below that spot diffusion starts at the spatial position $\boldsymbol{x}_0 = (x, y) = (0, 0)$. Then, we have:

$$\begin{aligned}
(h * C)(x, y, t) &= \int_{-\infty}^{+\infty}\int_{-\infty}^{+\infty} C(r, s, t)\, h(x - r, y - s)\, \mathrm{d}r\, \mathrm{d}s \\
&= \int_{-\infty}^{+\infty}\int_{-\infty}^{+\infty} \frac{C_0}{4\pi(t - t_0)D} \exp\left(-\frac{r^2 + s^2}{4(t - t_0)D}\right) \frac{1}{2\pi\sigma_{\text{PSF}}^2} \exp\left(-\frac{(x - r)^2 + (y - s)^2}{2\sigma_{\text{PSF}}^2}\right) \mathrm{d}r\, \mathrm{d}s \\
&= \frac{C_0}{8\pi^2(t - t_0)D\sigma_{\text{PSF}}^2} \exp\left(-\frac{x^2 + y^2}{2\sigma_{\text{PSF}}^2}\right) \int_{-\infty}^{+\infty}\int_{-\infty}^{+\infty} \exp\left(-\frac{r^2 + s^2}{4(t - t_0)D} - \frac{r^2 - 2xr + s^2 - 2ys}{2\sigma_{\text{PSF}}^2}\right) \mathrm{d}r\, \mathrm{d}s\,.
\end{aligned}$$

Denote

$$A = \frac{C_0}{8\pi^2(t - t_0)D\sigma_{\text{PSF}}^2}, \quad a = \frac{1}{2\sigma_{\text{PSF}}^2}\sqrt{\frac{4(t - t_0)D\sigma_{\text{PSF}}^2}{2(t - t_0)D + \sigma_{\text{PSF}}^2}}, \quad b = \sqrt{\frac{2(t - t_0)D + \sigma_{\text{PSF}}^2}{4(t - t_0)D\sigma_{\text{PSF}}^2}}\,.$$

Then, we have

$$\begin{aligned}
(h * C)(x, y, t) &= A\, e^{-\frac{x^2 + y^2}{2\sigma_{\text{PSF}}^2}} \int_{-\infty}^{+\infty}\int_{-\infty}^{+\infty} e^{-(br - ax)^2 + a^2x^2}\, e^{-(bs - ay)^2 + a^2y^2}\, \mathrm{d}r\, \mathrm{d}s \\
&= A\, e^{-\frac{x^2 + y^2}{2\sigma_{\text{PSF}}^2}}\, e^{a^2(x^2 + y^2)} \int_{-\infty}^{+\infty}\int_{-\infty}^{+\infty} e^{-(br - ax)^2}\, e^{-(bs - ay)^2}\, \mathrm{d}r\, \mathrm{d}s \\
&= A\, e^{-(x^2 + y^2)(\frac{1}{2\sigma_{\text{PSF}}^2} - a^2)} \int_{-\infty}^{+\infty}\int_{-\infty}^{+\infty} e^{-(br - ax)^2}\, e^{-(bs - ay)^2}\, \mathrm{d}r\, \mathrm{d}s \\
&= A\, e^{-(x^2 + y^2)(\frac{1}{2\sigma_{\text{PSF}}^2} - a^2)} \int_{-\infty}^{+\infty} e^{-(bs - ay)^2} \left(\int_{-\infty}^{+\infty} e^{-(br - ax)^2}\, \mathrm{d}r\right) \mathrm{d}s.
\end{aligned}$$

Denote $R = br - ax$ and $S = bs - ay$. Then, $\frac{dR}{dr} = b$ and $\frac{dS}{ds} = b$. It follows that

$$\begin{aligned}
(h * C)(x, y, t) &= A \exp\left(-(x^2 + y^2)\left(\frac{1}{2\sigma_{\text{PSF}}^2} - a^2\right)\right) \int_{-\infty}^{+\infty} e^{-S^2} \left(\int_{-\infty}^{+\infty} e^{-R^2}\, \mathrm{d}R\right) \mathrm{d}S \\
&= A \exp\left(-(x^2 + y^2)\left(\frac{1}{2\sigma_{\text{PSF}}^2} - a^2\right)\right) \int_{-\infty}^{+\infty} e^{-S^2} \times \frac{\sqrt{\pi}}{b}\, \mathrm{d}S \\
&= A \exp\left(-(x^2 + y^2)\left(\frac{1}{2\sigma_{\text{PSF}}^2} - a^2\right)\right) \frac{\pi}{b^2}
\end{aligned}$$

$$= \frac{4\pi C_0(t-t_0)D\sigma_{\text{PSF}}^2}{8\pi^2(t-t_0)D\sigma_{\text{PSF}}^2\left(2(t-t_0)D+\sigma_{\text{PSF}}^2\right)}\exp\left(-(x^2+y^2)\left(\frac{1}{2\sigma_{\text{PSF}}^2}-a^2\right)\right)$$

$$= \frac{C_0}{2\pi\left(2(t-t_0)D+\sigma_{\text{PSF}}^2\right)}\exp\left(-\left(x^2+y^2\right)\left(\frac{1}{2\sigma_{\text{PSF}}^2}-\frac{1}{4\sigma_{\sigma_{\text{PSF}}}^4}\frac{4(t-t_0)D\sigma_{\text{PSF}}^2}{2(t-t_0)D+\sigma_{\text{PSF}}^2}\right)\right)$$

$$= \frac{C_0}{2\pi\left(2(t-t_0)D+\sigma_{\text{PSF}}^2\right)}\exp\left(-\frac{x^2+y^2}{4(t-t_0)D+2\sigma_{\text{PSF}}^2}\right).$$

The fluorescence intensity is then of the following form:

$$f(x,y,t) = B\times\frac{C_0}{2\pi\left(2(t-t_0)D+\sigma_{\text{PSF}}^2\right)}\times\exp\left(-\frac{x^2+y^2}{4(t-t_0)D+2\sigma_{\text{PSF}}^2}\right).$$

Finally, the expression of intensity at spatial position $\boldsymbol{x}$ given an initial spot position located at $\boldsymbol{x}_0$ is given by :

$$\boxed{f(\boldsymbol{x},t) = \frac{A_0}{2D(t-t_0)+\sigma_{\text{PSF}}^2}\exp\left(-\frac{\|\boldsymbol{x}-\boldsymbol{x}_0\|_2^2}{4D(t-t_0)+2\sigma_{\text{PSF}}^2}\right),}$$

with $A_0 = C_0B/2\pi$.

B. Calculation of the autocorrelation G_1 in the case of uniform background

Let $f(\boldsymbol{x},t)$ be the fluorescent intensity at point $\boldsymbol{x}$ and time t with $\boldsymbol{x} \in \Omega$, $\Omega \subset \mathbb{R}^2$ and $t \in \mathbb{R}^+$. We denote $f(t)$ the t-th image in the sequence. The intensity at spatial position $\boldsymbol{x}$ of diffusing spot initially located at $\boldsymbol{x}_0$ and starting diffusion time $t = t_0$ is as follows:

$$f(\boldsymbol{x},t) \;=\; \frac{A_0}{2D(t-t_0)+\sigma^2_{\mathrm{PSF}}} \exp\left(-\frac{\|\boldsymbol{x}-\boldsymbol{x}_0\|_2^2}{4D(t-t_0)+2\sigma^2_{\mathrm{PSF}}}\right), \tag{2}$$

Let us consider the following autocorrelation function:

$$G_1(t,\tau) = \frac{\langle f(\boldsymbol{x},t) f(\boldsymbol{x},t+\tau)\rangle}{\langle f\rangle_t^2},$$

where τ is the time lag and $\langle\cdot\rangle$ denotes the spatial mean. The autocorrelation G_1 can be expressed in the case of diffusing spot as follows given (2) and $t_0 = 0$ (without loss of generality):

$$G_1(t,\tau) \;=\; \frac{\left\langle \dfrac{A_0^2}{(2Dt+\sigma^2_{\mathrm{PSF}})(2D(t+\tau)+\sigma^2_{\mathrm{PSF}})} \exp\left(-\dfrac{\|\boldsymbol{x}-\boldsymbol{x}_0\|_2^2}{4Dt+2\sigma^2_{\mathrm{PSF}}} - \dfrac{\|\boldsymbol{x}-\boldsymbol{x}_0\|_2^2}{4D(t+\tau)+2\sigma^2_{\mathrm{PSF}}}\right)\right\rangle}{\left\langle \dfrac{A_0}{2Dt+\sigma^2_{\mathrm{PSF}}} \exp\left(-\dfrac{\|\boldsymbol{x}-\boldsymbol{x}_0\|_2^2}{4Dt+2\sigma^2_{\mathrm{PSF}}}\right)\right\rangle^2}$$

Denote $\sigma^2(t) = 2Dt + \sigma^2_{\mathrm{PSF}}$. Hence, we have

$$\begin{aligned}
G_1(t,\tau) \;&=\; \frac{\left\langle \dfrac{A_0^2}{\sigma^2(t)\sigma^2(t+\tau)} \exp\left(-\dfrac{\|\boldsymbol{x}-\boldsymbol{x}_0\|_2^2}{2\sigma^2(t)} - \dfrac{\|\boldsymbol{x}-\boldsymbol{x}_0\|_2^2}{2\sigma^2(t+\tau)}\right)\right\rangle}{\left\langle \dfrac{A_0}{\sigma^2(t)} \exp\left(-\dfrac{\|\boldsymbol{x}-\boldsymbol{x}_0\|_2^2}{2\sigma^2(t)}\right)\right\rangle^2} \\
&=\; \frac{A_0^2}{\sigma^2(t)\sigma^2(t+\tau)} \, \frac{\sigma^4(t)}{A_0^2} \, \frac{\left\langle \exp\left(-\dfrac{\|\boldsymbol{x}-\boldsymbol{x}_0\|_2^2\left(\sigma^2(t)+\sigma^2(t+\tau)\right)}{2\sigma^2(t)\sigma^2(t+\tau)}\right)\right\rangle}{\left\langle \exp\left(-\dfrac{\|\boldsymbol{x}-\boldsymbol{x}_0\|_2^2}{2\sigma^2(t)}\right)\right\rangle^2} \\
&=\; \frac{\sigma^2(t)}{\sigma^2(t+\tau)} \, \frac{\left\langle \exp\left(-\dfrac{\|\boldsymbol{x}-\boldsymbol{x}_0\|_2^2\left(\sigma^2(t)+\sigma^2(t+\tau)\right)}{2\sigma^2(t)\sigma^2(t+\tau)}\right)\right\rangle}{\left\langle \exp\left(-\dfrac{\|\boldsymbol{x}-\boldsymbol{x}_0\|_2^2}{2\sigma^2(t)}\right)\right\rangle^2} \\
&=\; \frac{\sigma^2(t)}{\sigma^2(t+\tau)} \, \frac{\dfrac{1}{|\Omega|}\displaystyle\int_{\boldsymbol{x}\in\Omega} \exp\left(-\dfrac{\|\boldsymbol{x}-\boldsymbol{x}_0\|_2^2\left(\sigma^2(t)+\sigma^2(t+\tau)\right)}{2\sigma^2(t)\sigma^2(t+\tau)}\right) \mathrm{d}\boldsymbol{x}}{\left[\dfrac{1}{|\Omega|}\displaystyle\int_{\boldsymbol{x}\in\Omega} \exp\left(-\dfrac{\|\boldsymbol{x}-\boldsymbol{x}_0\|_2^2}{2\sigma^2(t)}\right) \mathrm{d}\boldsymbol{x}\right]^2} \\
&=\; |\Omega| \, \frac{\sigma^2(t)}{\sigma^2(t+\tau)} \, \frac{\displaystyle\int_{\boldsymbol{x}\in\Omega} \exp\left(-\dfrac{\|\boldsymbol{x}-\boldsymbol{x}_0\|_2^2\left(\sigma^2(t)+\sigma^2(t+\tau)\right)}{2\sigma^2(t)\sigma^2(t+\tau)}\right) \mathrm{d}\boldsymbol{x}}{\left[\displaystyle\int_{\boldsymbol{x}\in\Omega} \exp\left(-\dfrac{\|\boldsymbol{x}-\boldsymbol{x}_0\|_2^2}{2\sigma^2(t)}\right) \mathrm{d}\boldsymbol{x}\right]^2}.
\end{aligned}$$

As the integral of the exponential on the domain Ω is approximately equal to the integral on $\mathbb{R}^2$ when $\boldsymbol{x}$ is far from $\boldsymbol{x}_0$, which results in a Gauss integral. Finally, we have:

$$\begin{aligned} G_1(t,\tau) &\approx |\Omega| \frac{\sigma^2(t)}{\sigma^2(t+\tau)} \frac{2\pi\,\sigma^2(t)\sigma^2(t+\tau)}{\sigma^2(t)+\sigma^2(t+\tau)} \frac{1}{\left[2\pi\,\sigma^2(t)\right]^2} \approx \frac{|\Omega|}{2\pi\,\left(\sigma^2(t)+\sigma^2(t+\tau)\right)} \\ &\approx \frac{|\Omega|}{4\pi\left(D\tau+2Dt+\sigma^2_{\mathrm{PSF}}\right)} \end{aligned}$$

It turns out that the autocorrelation function depends on both the time lag τ and time t unlike the usual equations of TICS which assume temporal stationarity of the diffusing process.

C. Calculation of the autocorrelation G_2 in the case of non-uniform background

In the case of non-uniform background, the assumption that the value of the mean fluorescent intensity is zero after a long enough time does not hold. In order to compute the autocorrelation function G_2, the mean value of the fluorescent intensity has to be taken into account as follows:

$$G_2(t,\tau) = \frac{\langle\, \left(f(\boldsymbol{x},t) - \bar{f}\right)\left(f(\boldsymbol{x},t+\tau) - \bar{f}\right)\,\rangle}{\langle\, f\,\rangle_t^2}$$

where $\bar{f} = \frac{1}{T-t}\int_t^T f(\boldsymbol{x},\theta)\,\mathrm{d}\theta$. The explicit formulation of $G_2(\tau)$ is then derived as follows:

$$G_2(t,\tau) \;=\; \frac{\langle\, f(\boldsymbol{x},t)f(\boldsymbol{x},t+\tau)\,\rangle}{\langle\, f\,\rangle_t^2} - \frac{\langle\, f(\boldsymbol{x},t+\tau)\bar{f}\,\rangle}{\langle\, f\,\rangle_t^2} - \frac{\langle\, f(\boldsymbol{x},t)\bar{f}\,\rangle}{\langle\, f\,\rangle_t^2} + \frac{\langle\, \bar{f}_t^2\,\rangle}{\langle\, f\,\rangle_t^2}. \tag{3}$$

We calculate separately all the terms involved in (3). The first term is the expression of G_1 (see Appendix B):

$$\frac{\langle\, f(\boldsymbol{x},t)f(\boldsymbol{x},t+\tau)\,\rangle}{\langle\, f\,\rangle_t^2} \;=\; G_1(t,\tau) \;\approx\; \frac{|\Omega|}{4\pi\left(D\tau + 2Dt + \sigma_{\mathrm{PSF}}^2\right)} \tag{4}$$

The second term is of the following form:

$$\begin{aligned}
\langle\, f(\boldsymbol{x},t+\tau)\bar{f}\,\rangle \;&=\; \left\langle \frac{A_0}{2D(t+\tau)+\sigma_{\mathrm{PSF}}^2}\exp\left(-\frac{\|\boldsymbol{x}-\boldsymbol{x}_0\|_2^2}{4D(t+\tau)+2\sigma_{\mathrm{PSF}}^2}\right)\frac{1}{T-t}\int_t^T f(\boldsymbol{x},\theta)\mathrm{d}\theta \right\rangle \\
&=\; \frac{A_0}{(2D(t+\tau)+\sigma_{\mathrm{PSF}}^2)(T-t)}\frac{1}{|\Omega|}\int_{\boldsymbol{x}\in\Omega}\exp\left(-\frac{\|\boldsymbol{x}-\boldsymbol{x}_0\|_2^2}{4D(t+\tau)+2\sigma_{\mathrm{PSF}}^2}\right)\int_t^T f(\boldsymbol{x},\theta)\mathrm{d}\theta\,\mathrm{d}\boldsymbol{x} \\
&=\; \frac{A_0}{\sigma^2(t+\tau)(T-t)|\Omega|}\int_{\boldsymbol{x}\in\Omega}\int_t^T \exp\left(-\frac{\|\boldsymbol{x}-\boldsymbol{x}_0\|_2^2}{2\sigma^2(t+\tau)}\right)\frac{A_0}{\sigma^2(\theta)}\exp\left(-\frac{\|\boldsymbol{x}-\boldsymbol{x}_0\|_2^2}{2\sigma^2(\theta)}\right)\mathrm{d}\theta\,\mathrm{d}\boldsymbol{x}
\end{aligned}$$

where $\sigma^2(t) = 2Dt + \sigma_{\mathrm{PSF}}^2$. By inverting the two integrals, using the Gauss integral (see Appendix A), as well as by applying changes of variables (e.g., $x = D(\theta + t + \sigma) + \sigma_{\mathrm{PSF}}^2$, $\mathrm{d}x = D\mathrm{d}\theta$), one obtains

$$\langle\, f(\boldsymbol{x},t+\tau)\bar{f}\,\rangle \;=\; \frac{\pi A_0^2}{D|\Omega|(T-t)}\log\left(\frac{D(t+T+\tau)+\sigma_{\mathrm{PSF}}^2}{D(2t+\tau)+\sigma_{\mathrm{PSF}}^2}\right). \tag{5}$$

In the same manner, one obtains

$$\langle\, f(\boldsymbol{x},t)\bar{f}\,\rangle \;=\; \frac{\pi A_0^2}{D|\Omega|(T-t)}\log\left(\frac{D(t+T)+\sigma_{\mathrm{PSF}}^2}{2Dt+\sigma_{\mathrm{PSF}}^2}\right). \tag{6}$$

The calculation of $\langle\bar{f}^2\,\rangle$ is as follows:

$$\begin{aligned}
\langle\bar{f}\,\bar{f}\,\rangle \;&=\; \left\langle \frac{1}{(T-t)^2}\int_t^T f(\boldsymbol{x},\theta_1)\mathrm{d}\theta_1 \int_t^T f(\boldsymbol{x},\theta_2)\mathrm{d}\theta_2 \right\rangle \\
&=\; \frac{1}{|\Omega|(T-t)^2}\int_{\boldsymbol{x}\in\Omega}\int_t^T\int_t^T f(\boldsymbol{x},\theta_1)f(\boldsymbol{x},\theta_2)\,\mathrm{d}\theta_1\,\mathrm{d}\theta_2\,\mathrm{d}\boldsymbol{x} \\
&=\; \frac{1}{|\Omega|(T-t)^2}\int_{\boldsymbol{x}\in\Omega}\int_t^T\int_t^T \frac{A_0}{2D\theta_1+\sigma_{\mathrm{PSF}}^2}\exp\left(-\frac{\|\boldsymbol{x}-\boldsymbol{x}_0\|_2^2}{4D\theta_1+2\sigma_{\mathrm{PSF}}^2}\right)\frac{A_0}{2D\theta_2+\sigma_{\mathrm{PSF}}^2}\exp\left(-\frac{\|\boldsymbol{x}-\boldsymbol{x}_0\|_2^2}{4D\theta_2+2\sigma_{\mathrm{PSF}}^2}\right)\mathrm{d}\theta_1\,\mathrm{d}\theta_2\,\mathrm{d}\boldsymbol{x} \\
&=\; \frac{A_0^2}{|\Omega|(T-t)^2}\int_{\boldsymbol{x}\in\Omega}\int_t^T\int_t^T \frac{1}{\sigma^2(\theta_1)}\exp\left(-\frac{\|\boldsymbol{x}-\boldsymbol{x}_0\|_2^2}{2\sigma^2(\theta_1)}\right)\frac{1}{\sigma^2(\theta_2)}\exp\left(-\frac{\|\boldsymbol{x}-\boldsymbol{x}_0\|_2^2}{2\sigma^2(\theta_2)}\right)\mathrm{d}\theta_1\,\mathrm{d}\theta_2\,\mathrm{d}\boldsymbol{x}
\end{aligned}$$

where $\sigma^2(\theta) = 2D\theta + \sigma^2_{\text{PSF}}$. By inverting the integrals, using the Gauss integral, as well as by applying a triple change of variables: $x = D(\theta_1 + \theta_2) + \sigma^2_{\text{PSF}}$, $\mathrm{d}x = D\mathrm{d}\theta_1$, then $y = D(t + \theta_2) + \sigma^2_{\text{PSF}}$, $\mathrm{d}y = D\mathrm{d}\theta_2$ and then $z = y + D(T - t)$, $\mathrm{d}z = \mathrm{d}y$) one obtains

$$\langle \bar{f}\,\bar{f}\rangle = \frac{\pi A_0^2}{D^2|\Omega|(T-t)^2}\left[\left(2DT + \sigma^2_{\text{PSF}}\right)\log\left(\frac{2DT + \sigma^2_{\text{PSF}}}{D(T+t) + \sigma^2_{\text{PSF}}}\right) + \left(2Dt + \sigma^2_{\text{PSF}}\right)\log\left(\frac{2Dt + \sigma^2_{\text{PSF}}}{D(T+t) + \sigma^2_{\text{PSF}}}\right)\right]. \tag{7}$$

The last term to calculate is

$$\langle f\rangle_t^2 = \left\langle \frac{A_0}{2Dt + \sigma^2_{\text{PSF}}}\exp\left(-\frac{\|\boldsymbol{x} - \boldsymbol{x}_0\|_2^2}{4Dt + 2\sigma^2_{\text{PSF}}}\right)\right\rangle^2 = \left[\int_{\boldsymbol{x}\in\Omega}\frac{A_0}{2Dt + \sigma^2_{\text{PSF}}}\exp\left(-\frac{\|\boldsymbol{x} - \boldsymbol{x}_0\|_2^2}{4Dt + 2\sigma^2_{\text{PSF}}}\right)\mathrm{d}\boldsymbol{x}\right]^2$$

$$= \left[\int_{\boldsymbol{x}\in\Omega}\frac{A_0}{\sigma^2(t)}\exp\left(-\frac{\|\boldsymbol{x} - \boldsymbol{x}_0\|_2^2}{2\sigma^2(t)}\right)\mathrm{d}\boldsymbol{x}\right]^2$$

As before, the integral of the exponential is the Gauss integral, which yields

$$\langle f\rangle_t^2 = \frac{4\pi^2 A_0^2}{|\Omega|^2}. \tag{8}$$

Finally, the autocorrelation model G_2 obtained by substituting the terms (4)-(8) into (3):

$$G_2(t,\tau) = G_1(\tau) - \frac{\frac{\pi A_0^2}{D|\Omega|(T-t)}\log\left(\frac{D(t+T+\tau)+\sigma^2_{\text{PSF}}}{D(2t+\tau)+\sigma^2_{\text{PSF}}}\right)}{\frac{4\pi^2 A_0^2}{|\Omega|^2}} - \frac{\frac{\pi A_0^2}{D|\Omega|(T-t)}\log\left(\frac{D(t+T)+\sigma^2_{\text{PSF}}}{2Dt+\sigma^2_{\text{PSF}}}\right)}{\frac{4\pi^2 A_0^2}{|\Omega|^2}}$$
$$+ \frac{\frac{\pi A_0^2}{D^2|\Omega|(T-t)^2}\left[\left(2DT + \sigma^2_{\text{PSF}}\right)\log\left(\frac{2DT+\sigma^2_{\text{PSF}}}{D(T+t)+\sigma^2_{\text{PSF}}}\right) + \left(2Dt + \sigma^2_{\text{PSF}}\right)\log\left(\frac{2Dt+\sigma^2_{\text{PSF}}}{D(T+t)+\sigma^2_{\text{PSF}}}\right)\right]}{\frac{4\pi^2 A_0^2}{|\Omega|^2}}$$

After all simplifications, one gets

$$G_2(t,\tau) = G_1(t,\tau) + |\Omega|\left[\frac{1}{4\pi D(T-t)}\log\left(\frac{D\tau + 2Dt + \sigma^2_{\text{PSF}}}{D\tau + D(T+t) + \sigma^2_{\text{PSF}}}\right)\right.$$
$$\left. + \frac{2DT + \sigma^2_{\text{PSF}}}{4\pi D^2(T-t)^2}\log\left(\frac{2DT + \sigma^2_{\text{PSF}}}{D(T+t) + \sigma^2_{\text{PSF}}}\right) + \frac{D(T+t) + \sigma^2_{\text{PSF}}}{4\pi D^2(T-t)^2}\log\left(\frac{2Dt + \sigma^2_{\text{PSF}}}{D(T+t) + \sigma^2_{\text{PSF}}}\right)\right].$$

If we denote

$$K_1(t,\tau) = \frac{1}{4\pi D(T-t)}\log\left(\frac{D\tau + 2Dt + \sigma^2_{\text{PSF}}}{D\tau + D(T+t) + \sigma^2_{\text{PSF}}}\right),$$

$$K_2(\tau) = \frac{2DT + \sigma^2_{\text{PSF}}}{4\pi D^2(T-t)^2}\log\left(\frac{2DT + \sigma^2_{\text{PSF}}}{D(T+t) + \sigma^2_{\text{PSF}}}\right) + \frac{D(T+t) + \sigma^2_{\text{PSF}}}{4\pi D^2(T-t)^2}\log\left(\frac{2Dt + \sigma^2_{\text{PSF}}}{D(T+t) + \sigma^2_{\text{PSF}}}\right),$$

it follows that

$$G_2(t,\tau) = G_1(t,\tau) + |\Omega|\left[K_1(t,\tau) + K_2(t)\right].$$

D. Influence of the ROI size and spot position in the ROI

In this section, we apply the BayesTICS algorithm on artificial image sequences to compute the MAP and MMSE estimators of the diffusion coefficient given model G_2 (9). Three scenarios were tested: varying ROI size, varying spot position in the ROI, and combined varying ROI size and spot position. We simulated a sequence of 256×256 pixels noise-free images depicting multiple diffusing spots, with a diffusion coefficient equal to 0.25 pixels/frame (see Supplementary Fig. 1**a**). The spots appear at random locations and at different times. Several test sequences corresponding to the three given scenarios were extracted from this main sequence. For each scenario, we describe the test sequences, then discuss the estimation results in terms of fitting as well as in terms of MAP and MMSE estimation of the diffusion coefficient.

D.0.1. Influence of the ROI size

In this scenario, six test sequences corresponding to six different ROIs were used. Each sequence is composed of 250 frames. As an illustration, four typical images are shown in Supplementary Fig. 1**b** at times $t = 0, 15, 20$ and 25. The spot of interest is assumed to be located at the center of the ROI. Time t = t_0 corresponds to the frame where this spot appears at the cell surface. The two secondary diffusing spots appear at $t_0 + 15$ and $t_0 + 20$, respectively. The largest ROI covers the spot of interest and the two other spots diffusing in the neighborhood (Supplementary Fig. 1**c**), while the smallest ROI only contains the spot of interest (Supplementary Fig. 1**h**).

We computed the MAP and MMSE estimators of the diffusion coefficient by increasing the ROI sizes as illustrated in Supplementary Fig. 1**c-h**. The autocorrelation fits very well for all ROI sizes, even though it appears to be even more precise for the smallest sizes. As for the MAP and MMSE estimators, they barely differ between sequences, with a similar influence of the ROI size on the accuracy of the estimation. For the three largest ROIs (Supplementary Fig. 1**c-d-e**), both MAP and MMSE slightly underestimate the diffusion coefficient, with $\hat{D}_{\text{MAP}}$ being in the range $[0.22, 0.23]$ and $\hat{D}_{\text{MMMSE}}$ in the range $[0.23, 0.24]$ for a ground truth $D_{\text{true}} = 0.25$. For the fourth ROI (Supplementary Fig. 1**f**), the MAP and MMSE estimators are almost perfect with $\hat{D}_{\text{MAP}} = 0.24$ and $\hat{D}_{\text{MMMSE}} = 0.25$. For the two smallest ROIs (Supplementary Fig. 1**g-h**), both MAP and MMSE slightly overestimate the diffusion coefficient, with $\hat{D}_{\text{MAP}} = 0.26$ and $\hat{D}_{\text{MMMSE}} = 0.27$ in both cases. In conclusion, the ROI size has almost no influence on the estimation of the diffusion coefficient. It still appears that the MAP estimator performs best for small ROI sizes, while the MMSE estimator performs best for medium ROI sizes. In the case of clouded ROIs, the MMSE estimator appears more accurate than the MAP estimator.

D.0.2. Influence of the spot position in the ROI

From the simulated main sequence, six different ROIs of the same size were chosen around the same spot of interest (see Supplementary Fig. 2). Each ROI may contain either the full spot or only a part of it (Supplementary Fig. 2**c**), and another diffusing spot may be present in the ROI (Supplementary Fig. 2**d** and 2**f**). As in the previous case, each sequence is composed of 250 frames.

We applied the BayesTICS algorithm and computed the MAP and MMSE estimators in each case. The autocorrelation fitting is essentially the same for the six test sequences, and is consistent with the scenario of a medium to large size ROI seen previously. The diffusion coefficient is slightly underestimated for both MAP and MMSE, as expected from the first scenario: $\hat{D}_{\text{MAP}}$ is in the range $[0.21, 0.23]$ and $\hat{D}_{\text{MMMSE}}$ is in the range $[0.22, 0.23]$ for a ground truth $D_{\text{true}} = 0.25$. In particular, the MMSE estimator is very consistent between all cases, with $\hat{D}_{\text{MMMSE}} = 0.23$ in five out of the six cases. The presence of another spot diffusing in the ROI (Supplementary Fig. 2**f**) seems to amplify the underestimation of the diffusion coefficient: in this case, $\hat{D}_{\text{MAP}} = 0.21$ and $\hat{D}_{\text{MMMSE}} = 0.22$, with $\hat{D}_{\text{MMMSE}}$ being slightly more accurate than $\hat{D}_{\text{MAP}}$. The results still clearly show that both the MAP and MMSE estimators are robust to spot position.

D.0.3. Combined influence of the ROI size and spot position

Finally, it is important to evaluate the sensitivity of the MAP and MMSE estimators for both varying ROI size and varying spot location in the ROI. To address this, we extracted, from the simulated main sequence, the four test sequences illustrated in Supplementary Fig. 3. Various ROI sizes and ratios are considered. The spot of interest is placed in different locations, and several other spots, appearing after the spot of interest, are diffusing in the ROI. As before, each sequence is composed of 250 frames. Both the autocorrelation plots of these sequences, and the MAP and MMSE estimators computed with the BayesTICS method, are shown in Supplementary Fig. 3.

The autocorrelation fitting is consistent with what was observed in the previous scenarios. The estimation results are homogeneous for the last three cases, where $\hat{D}_{\text{MAP}}$ is in the range $[0.20, 0.21]$ and $\hat{D}_{\text{MMMSE}}$ is in the range

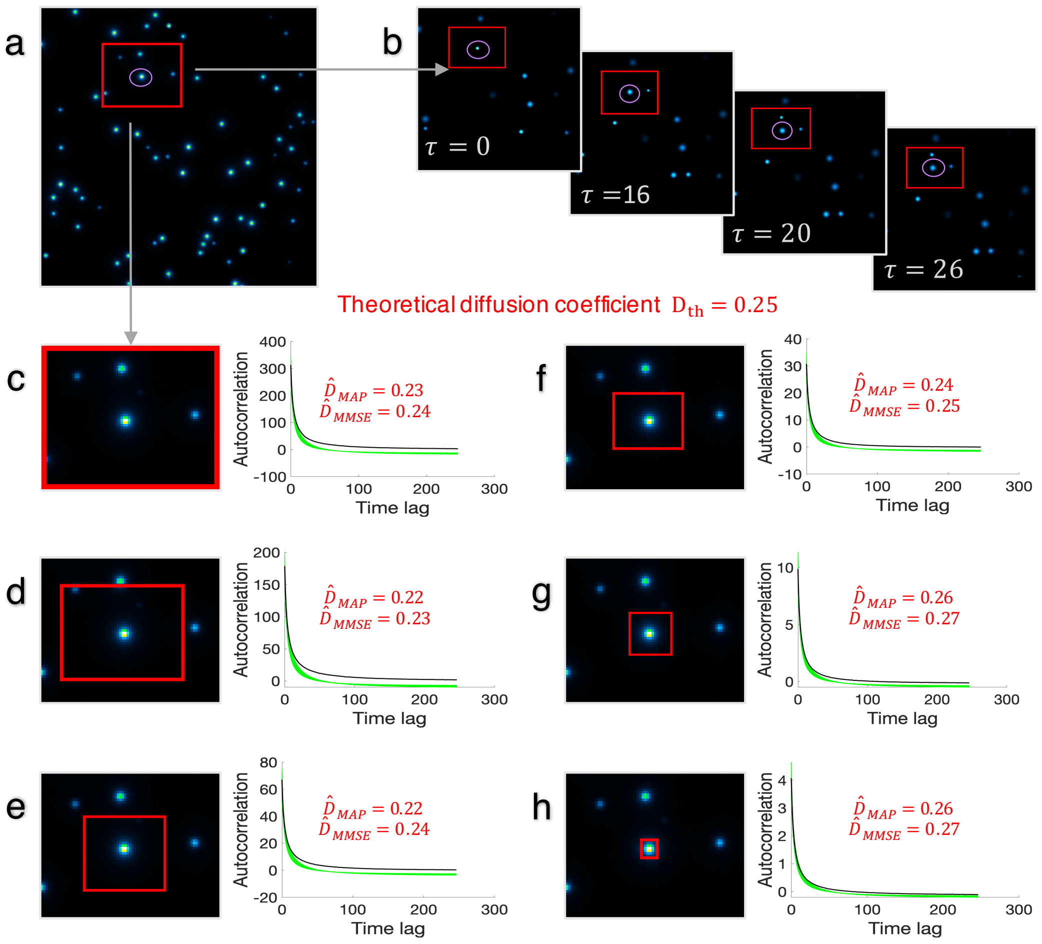

Supplementary Fig. 1. Robustness of BayesTICS to window size (with centered spot). a. *Simulated noise-free image sequence* (256 × 256 *pixels, 300 frames) of 2D diffusing spots with a theoretical diffusion coefficient of* D_{true} = 0.25 *pixels/frame. The displayed image is the maximum intensity projection of the 3D stack. The ROIs used for estimating the diffusion coefficient are shown in red, and the spot of interest in purple.* ***b.*** *Four images of the sequence are shown at time* $t = 0, 15, 20$ *and* 25*. The ROI and the spot of interest are also highlighted red (square) and purple (circle), respectively.* ***c-h.*** *Six ROI sizes were tested :* 73 × 57, 54 × 43, 31 × 28, 21 × 19, 12 × 11, *and* 8 × 7 *pixels windows. The ROI is centered on the spot of interest. Each plot shows the computed autocorrelation from the data (dark gray) and the generated autocorrelations corresponding to the estimated distribution of the diffusion coefficient from the BayesTICS method (green). The two estimates* $\hat{D}_{MAP}$ *and* $\hat{D}_{MMMSE}$ *are given for each window size on their respective plots.*

[0.21, 0.22]. The only result that seems significantly different is the one shown in Supplementary Fig. 3**b**, where $\hat{D}_{\mathrm{MAP}} = 0.17$ and $\hat{D}_{\mathrm{MMMSE}} = 0.19$. In this case, the fact that the ROI is clouded yields a significant underestimation of the diffusion coefficient for both MAP and MMSE estimators. From these results, we can conclude that the MAP and MMSE estimators are robust to the cumulative effect of the ROI size and spot position; however, caution is needed when handling large ROI sizes with a large number of other diffusing spots in the ROI.

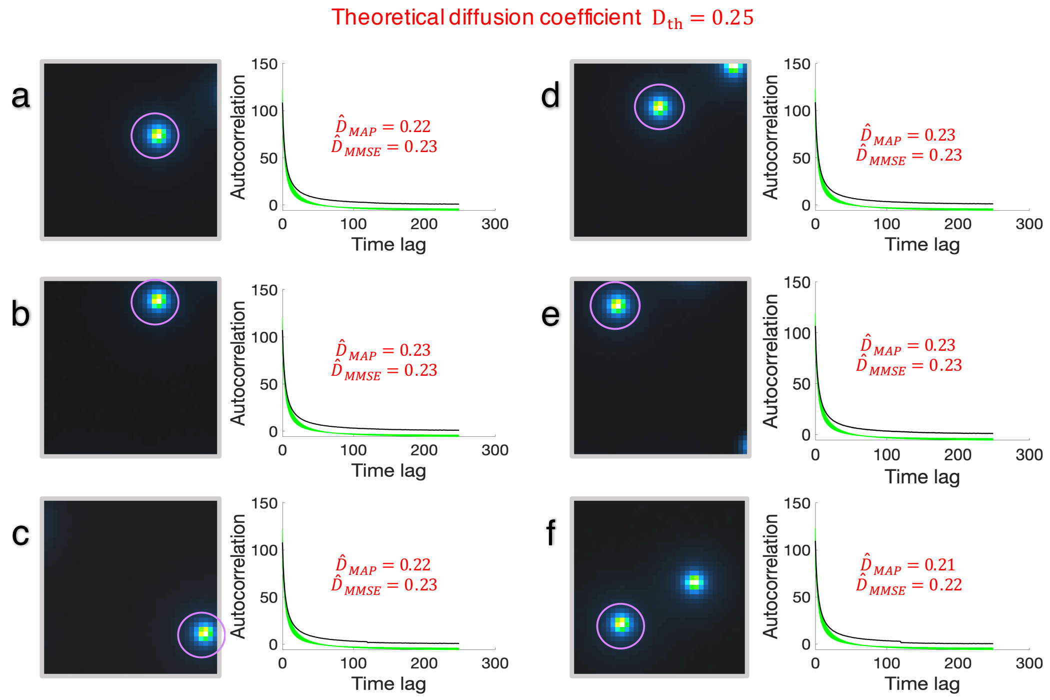

Supplementary Fig. 2. Robustness of BayesTICS to spot position (with fixed window size). a-f. *Six different cases were tested. Each ROI was extracted from a simulated image sequence (256 × 256 pixels, 300 frames) of 2D diffusing spots with a theoretical diffusion coefficient of* $D_{true} = 0.25$ *pixels/frame. For each ROI, we display the maximum intensity projection of the 3D stack and the plots of the autocorrelation versus time lag. The diffusing spot of interest is labeled in purple (circle). The spot can be located anywhere in the ROI. The ROI can contain supplementary spots diffusing potentially at the same time or not. The size of the ROI is fixed to 37 × 37 pixels. Each plot shows the computed autocorrelation from the data (dark gray) and the generated autocorrelations corresponding to the estimated distribution of the diffusion coefficient from the BayesTICS method (green). The two estimates* $\hat{D}_{MAP}$ *and* $\hat{D}_{MMMSE}$ *are given on the corresponding plots.*

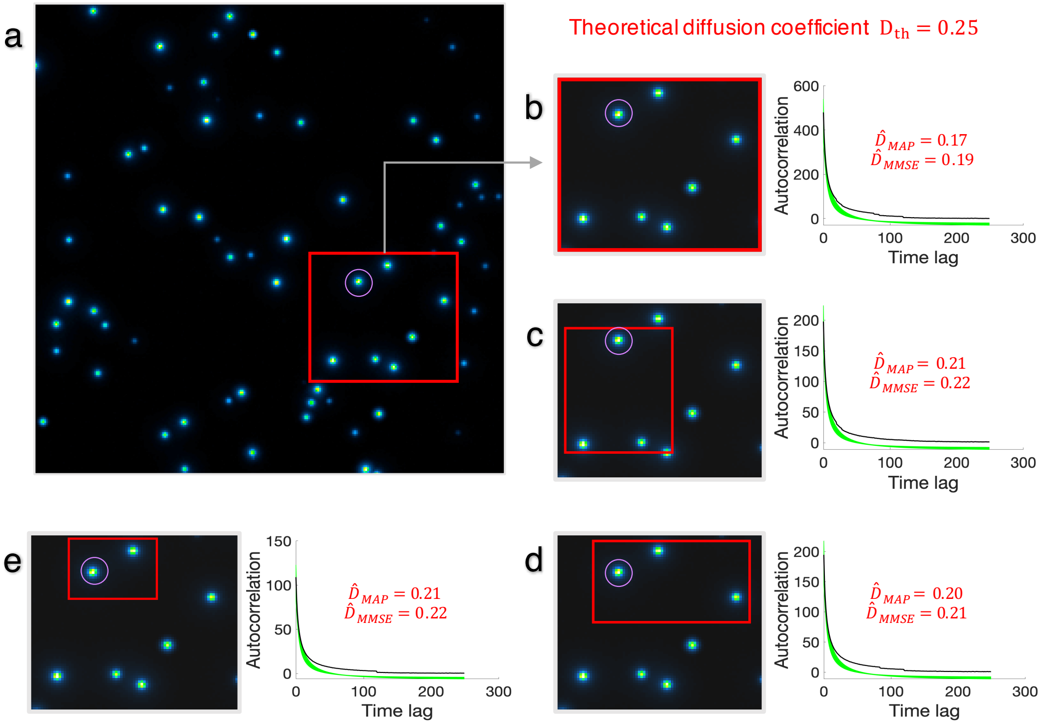

Supplementary Fig. 3. Robustness of BayesTICS to spot position with variable window size. a. *Maximum intensity projection of the 3D stack for the main simulated sequence (256 × 256 pixels, 300 frames) of 2D diffusing spots with a theoretical diffusion coefficient of* $D_{true} = 0.25$ *pixels/frame.* ***b-e.*** *Four different cases were tested, extracted from the main simulated sequence. For each ROI, we display the maximum intensity projection of the 3D stack and the plots of the autocorrelation versus time lag. The ROIs are shown in red (rectangle) and the spot of interest in purple (circle). The window size varies, from up to buttom and to the left, as following: 81 × 72, 49 × 51, 63 × 39, and 44 × 31 pixels. The spot of interest can be located anywhere in the ROI. The ROI can contain supplementary spots diffusing potentially at the same time or not. Each plot shows the computed autocorrelation from the data (dark gray) and the generated autocorrelations corresponding to the estimated distribution of the diffusion coefficient from the BayesTICS method (green). The two estimates* $\hat{D}_{MAP}$ *and* $\hat{D}_{MMMSE}$ *are given on the corresponding plots.*

E. BayesTICS applied to Rab11 proteins

In this section, we applied BayesTICS to TIRFM images depicting Rab11 proteins during the late stages of exocytosis. These experiments were performed in the context of a double labeling, Rab11-mCherry/Tfr-pHluorin. The videos of TfR and Rab11 proteins were jointly captured with a dual TIRF microscopy set-up. In this biological study, the primary idea is to characterize the phenomenon of vesicle fusion with the PM, diffusion of TfR at the PM, and dissociation of Rab11 after vesicle fusion with PM. Rab11 proteins are known to either dissociate in the cytosol after vesicle fusion or diffuse at the PM.[1]

A spot detection algorithm (see [4]) was applied to the TIRFM image sequence, which provided 378 spots potentially diffusing at the PM. The time-point t_0 of the beginning of fusion was estimated on the TfR spot. The burst effect in the fluorescent signal on this spot allows for the accurate estimation of t_0. Supplementary Fig. 4**a** illustrates two TfR and Rab11 spots having the same spatial and temporal coordinates. We then applied BayesTICS to all the detected spots. Finally, as BayesTICS is dedicated to diffusion estimation, we tested beforehand if the estimated posterior distribution for each spot corresponds to pure diffusion. The test was conducted as follows : first, we estimated the two parameters of a Gamma distribution that best fitted the posterior distribution provided by BayesTICS ; then, a Kolmogorov-Smirnov test was used to test the null hypothesis (with a 5% significance level) that the BayesTICS posterior distribution follows a Gamma distribution. With this procedure, we found that, between 2% and 11% of the Rab11 detected spots (depending on the chosen parameter D or σ_{PSF} and model G_1 or G_2) display apparent diffusion. Supplementary Fig. 4**b** illustrates the results for three Rab11 spots depicting apparent diffusion.

[1]Gidon, A *et al.* (2012). A Rab11A/myosin Vb/Rab11-FIP2 complex frames two late recycling steps of langerin from the ERC to the plasma membrane. *Traffic*, 13(6):815-833.

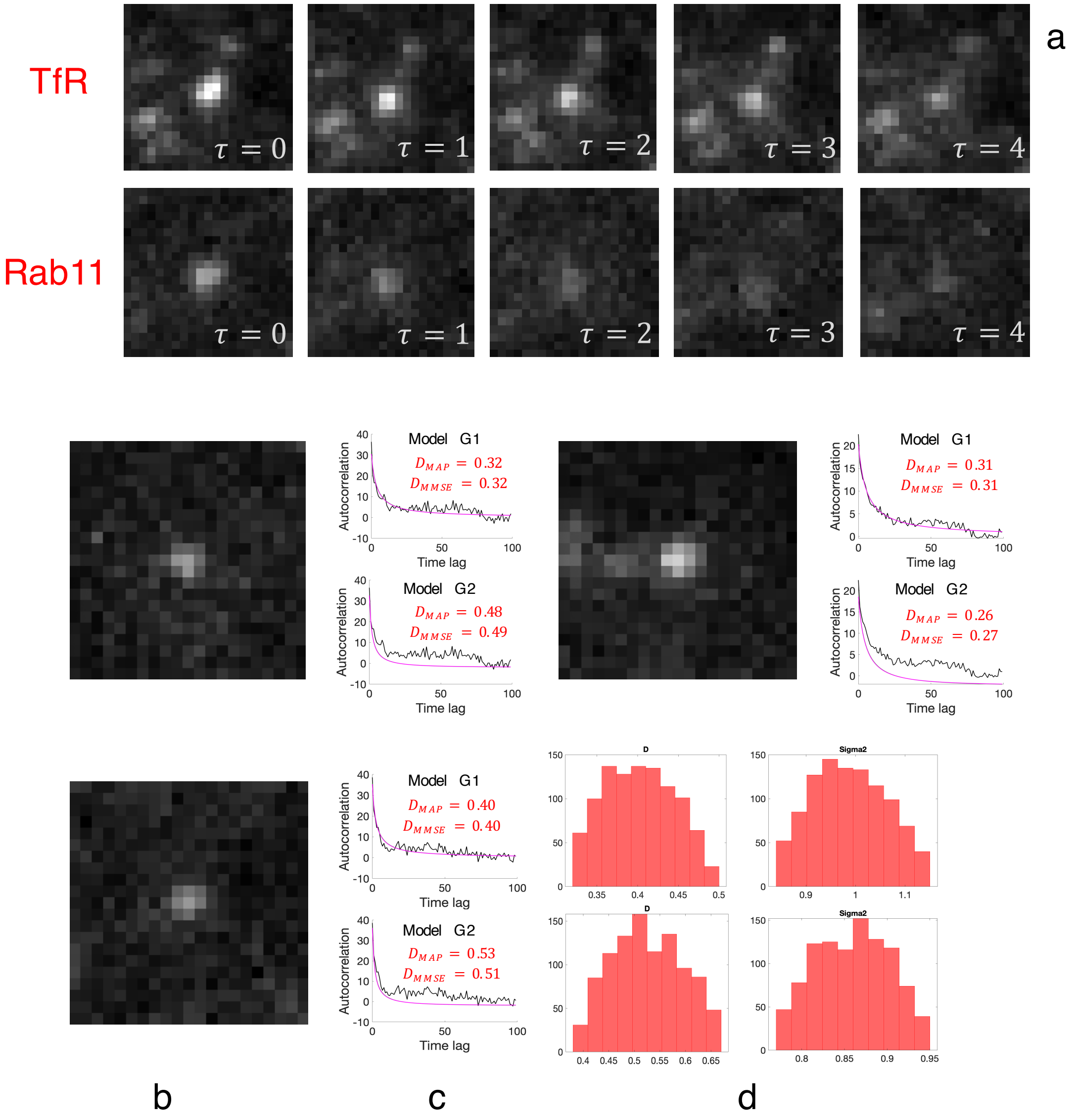

Supplementary Fig. 4. BayesTICS applied to Rab11 spots. a. *Illustration of five frames of a TfR spot (upper line), a Rab11 spot (lower line) displaying apparent diffusion and detected at the same spatial and temporal position.* ***b.*** *Illustration of the first frame for Rab11 spots.* ***c.*** *The autocorrelation versus time lag plot are shown for* G_1 *and* G_2 *models. Each plot shows the observed autocorrelation (black curve), and two autocorrelation samples generated from the* G_1 *and* G_2 *models with the* $\hat{D}_{MAP}$ *and* $\hat{D}_{MMMSE}$ *parameters (green and magenta curves).* ***d.*** *The estimated posterior distributions are displayed for D (left) and* σ_{PSF} *(right) for the third example of a Rab11 spot.*
